# Chronological age enhances aging phenomena and protein nitration in oocyte

**DOI:** 10.3389/fendo.2023.1251102

**Published:** 2023-12-12

**Authors:** Pravin T. Goud, Anuradha P. Goud, Olivia G. Camp, David Bai, Bernard Gonik, Michael P. Diamond, Husam M. Abu-Soud

**Affiliations:** ^1^ Laurel Fertility Center, San Francisco, CA, United States; ^2^ Division of Endocrinology, Diabetes and Metabolism, Department of Internal Medicine, University of California Davis Medical School, Sacramento, CA, United States; ^3^ Department of Obstetrics and Gynecology, University of California Davis Medical School, Sacramento, CA, United States; ^4^ Department of Obstetrics and Gynecology, The C.S. Mott Center for Human Growth and Development, Wayne State University School of Medicine, Detroit, MI, United States; ^5^ Division of Maternal Fetal Medicine, Department of Obstetrics and Gynecology, Wayne State University School of Medicine, Detroit, MI, United States; ^6^ Division of Reproductive Endocrinology and Infertility, Department of Obstetrics and Gynecology, Augusta University, Augusta, GA, United States; ^7^ Department of Physiology, Wayne State University School of Medicine, Detroit, MI, United States

**Keywords:** nitric oxide, oocyte aging, oocyte temporal window, oocyte quality, advanced reproductive age

## Abstract

**Background:**

The average age of childbearing has increased over the years contributing to infertility, miscarriages, and chromosomal abnormalities largely invoked by an age-related decline in oocyte quality. In this study, we investigate the role of nitric oxide (NO) insufficiency and protein nitration in oocyte chronological aging.

**Methods:**

Mouse oocytes were retrieved from young breeders (YB, 8-14 weeks [w]), retired breeders (RB, 48-52w) and old animals (OA, 80-84w) at 13.5 and 17 hours after ovulation trigger. They were assessed for zona pellucida dissolution time (ZPDT); ooplasmic microtubule dynamics (OMD); cortical granule (CG) status and spindle morphology (SM), as markers of oocyte quality. Sibling oocytes from RB were exposed to NO supplementation and assessed for aging phenomena (AP). All oocyte cumulus complexes were subjected to fluorescence nitrotyrosine (NT) immunocytochemistry and confocal microscopy to assess morphology and protein nitration.

**Results:**

At 13.5 h from hCG trigger, oocytes from RB compared to YB had significantly increased ZPDT (37.8 ± 11.9 vs 22.1 ± 4.1 seconds [s]), OMD (46.9 vs 0%), CG loss (39.4 vs 0%), and decreased normal SM (30.3 vs 81.3%), indicating premature AP that worsened among oocytes from RB at 17 hours post-hCG trigger. When exposed to SNAP, RB AP significantly decreased (ZPDT: 35.1 ± 5.5 vs 46.3 ± 8.9s, OMD: 13.3 vs 75.0% and CG loss: 50.0 vs 93.3%) and SM improved (80.0 vs 14.3%). The incidence of NT positivity was significantly higher in cumulus cells (13.5 h, 46.7 ± 4.5 vs 3.4 ± 0.7%; 17 h, 82.2 ± 2.9 vs 23.3 ± 3.6%) and oocytes (13.5 h, 57.1 vs 0%; 17 h, 100.0 vs 55.5%) from RB compared to YB. Oocytes retrieved decreased with advancing age (29.8 ± 4.1 per animal in the YB group compared to 10.2 ± 2.1 in RB and 4.0 ± 1.6 in OA). Oocytes from OA displayed increased ZPDT, major CG loss, increased OMD and spindle abnormalities, as well as pronuclear formation, confirming spontaneous meiosis to interphase transition.

**Conclusion(s):**

Oocytes undergo zona pellucida hardening, altered spindle and ooplasmic microtubules, and premature cortical granule release, indicative of spontaneous meiosis-interphase transition, as a function of chronological aging. These changes are also associated with NO insufficiency and protein nitration and may be alleviated through supplementation with an NO-donor.

## Introduction

Infertility, defined as an inability to conceive despite unprotected intercourse for 1 year, is recognized as a common problem affecting at least 1 in 6 couples (1). Sociodemographic changes over years have led to women to delay childbearing to a relatively older age compared to their predecessors (2); therefore, it is not uncommon to see women in mid- or later thirties or even later, trying to achieve pregnancy either for the first time or trying to grow their family. Thus, advanced age of the female partner has become an increasingly common contributing factor to infertility (3). Specifically, maternal age >36 years is frequently associated with a decline in the numbers of follicles within the ovaries, as well as deterioration of quality of oocytes within the follicles, resulting in reduced fecundity and increased reproductive loss (4–9). This is a significant problem despite the advent of assisted reproduction technologies (ART).

A wide array of endocrinological and related phenomena are noted among women during the years preceding menopause. These changes are mostly due to ovarian senescence related to depletion of follicles and endocrinological perturbations in the ovarian milieu (10–12). This is clinically reflected as shortened ovarian cycle length, altered size of cyclically recruited pool of follicles, and accelerated timing of dominant follicle selection and ovulation (13–19). Nonetheless such alterations may not fully explain structural and functional deterioration of oocytes from women >36 with regular ovulatory cycles. Such decreased fertility in women precedes characteristic reproductive aging during perimenopausal years and is likely related to specific alterations within the oocyte and its microenvironment within the follicle (3, 20, 21). Although the mechanisms involved in these alterations remain enigmatic, the specific nature is under investigation with the free radical theory of aging offering the most likely explanation. At low levels the production of reactive oxygen species (ROS) through cellular metabolism plays a physiological role; yet, as suggested by the mitochondrial free radical theory of aging, excess ROS may drive the aging process (22). Excess ROS may be mediating damage to mitochondrial DNA, leading to further ROS production, and creating a vicious cycle with eventual apoptosis. Other authors have suggested ROS are a responsive mechanism to the physiological changes associated with aging rather than a causative role, allowing for a stress response to age-dependent damage (22). Still, the involvement of ROS in oocyte quality deterioration is prevalent.

We have previously shown that nitric oxide (NO), a ubiquitous molecule in the oocyte microenvironment, is involved in sustaining oocyte quality and maintaining the optimal oocyte temporal window for fertilization (23, 24). On the other hand, reactive oxygen species (ROS) such as superoxide (O_2_^·-^), hydrogen peroxide (H_2_O_2_), and hypochlorous acid (HOCl) were all shown to cause specific phenomena in the oocytes characterized by hardening of the zona pellucida (ZP), accelerating premature cortical granule loss (CGL), ooplasmic microtubule enhancement (OME) in response to a microtubular enhancer, Taxol; as well as altered spindle morphology (SM) and chromosome alignment (11, 25). Importantly, oocytes retrieved from older animals were noted to have NO insufficiency, likely related to deficiency of substrates or cofactors involved in production of NO, as well as zinc (Zn) depletion (26). One other possibility is accelerated destruction or consumption of NO due to its interaction with ROS or mammalian peroxidases such as MPO, respectively (27).

In the current study, we address a hypothesis that chronological aging is associated with an insufficiency in NO and increased ROS in the oocyte microenvironment, and that an interplay between these factors results in deterioration in oocyte quality evident in the forms of characteristic changes in oocyte structure and function. In this report, a mouse model for chronological aging is used with young animals, retired breeders and old animals used to represent younger, middle aged and perimenopausal women.

## Methods

### Study design

**Figure 1:**
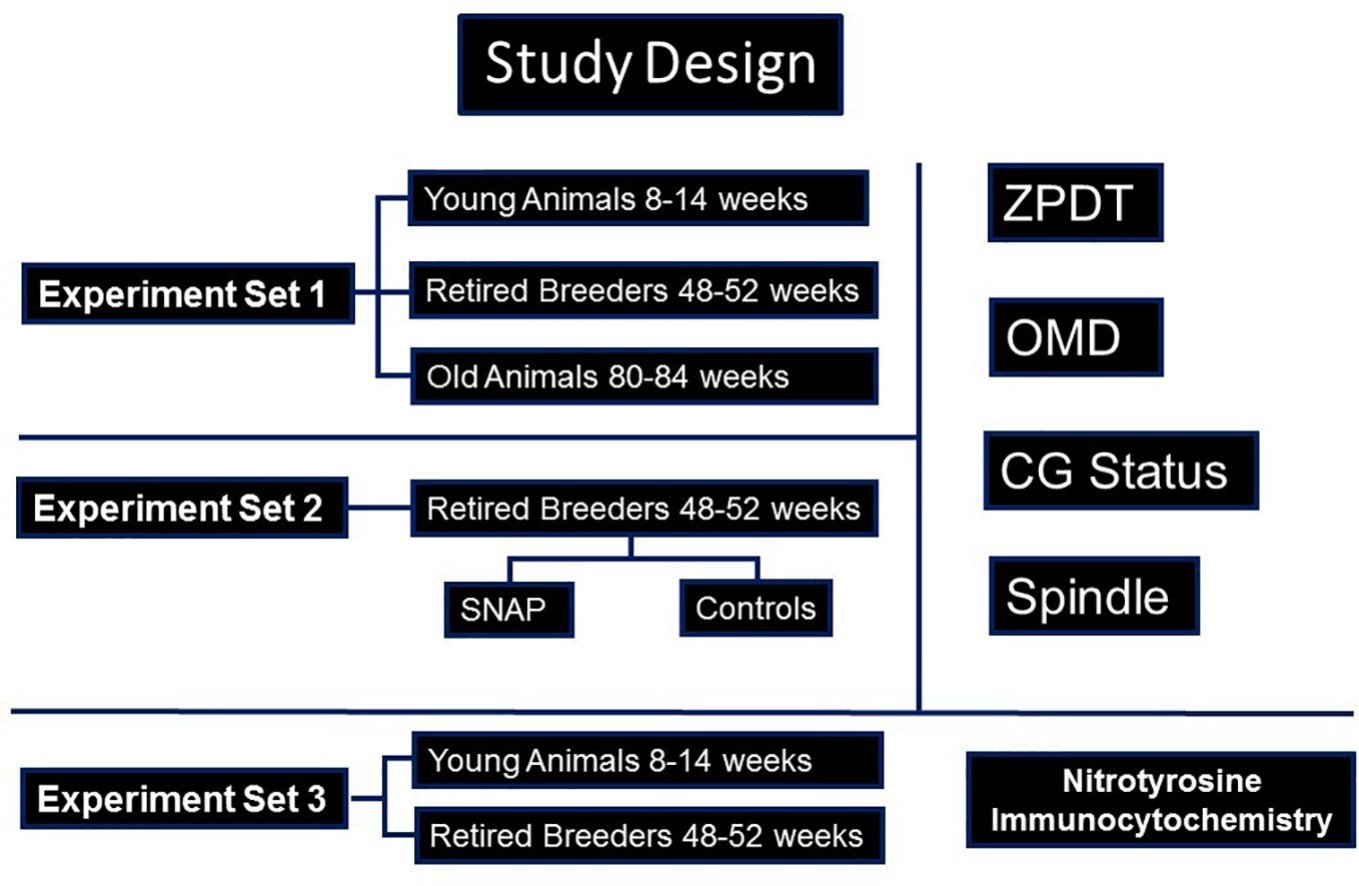
A flow-chart is presented, depicting the study design. As shown, three sets of experiments were performed. Accordingly, in experiment set 1, oocytes from young (YB) and retired breeders (RB), as well as old animals (OA) were assessed for cortical granules (CG), Spindles and ooplasmic microtubules (OM), as well as zona pellucida dissolution time (ZPDT), a parameter to assess zona pellucida hardening. In experiment set 2, sibling oocytes retrieved from RB were subjected to an NO-donor, SNAP, or its end product, penicillamine, prior to assessing aging phenomena. In experiment set 3, oocytes and cumulus cells were subjected to fluorescence immunocytochemistry for nitrotyrosine (NT).

The current study was approved by the institutional review board/Animal Investigations Committee of Wayne State University. All reagents were obtained from Sigma Aldritch (St Louis, MO) unless specified otherwise. Design of the study is presented in **Figure 1**. Due to their relatively shorter life-span as well as previous knowledge regarding the follicular dynamics and timing of ovarian senescence, we performed this study on oocytes from B6D2F1 female mice in three chronological age-groups (28–30). These groups comprised of mice aged 8-14 weeks (young breeders, YB), 48-52 weeks (retired breeders, RB), and 80-84 weeks (old animals, OA) since these age-groups are approximately comparable to young (<30 year old); middle aged (>36 year old) and older (>44 year old) women based on follicular dynamics and fecundability (28, 30–33). Mice from the three groups underwent superovulation with the help of PMSG and hCG, followed by retrieval of oviductal oocytes at 13.5 hours or 17 hours later, to study sustenance of oocyte quality post-ovulation (23). The oocytes were subjected to three sets of experiments (**Figure 1**). In experiment set 1, the oviductal oocytes were evaluated for determination of zona pellucida dissolution time (ZPDT), cortical granule (CG) status, spindle morphology (SM) and chromosome status as well as ooplasmic microtubule dynamics in response to Taxol (34–36). In experiment set 2, oocytes obtained from RB were retrieved at 13.5 hours and were treated with an NO donor, S-Nitroso-N-acetyl-DL-penicillamine (SNAP), or penicillamine (end product of SNAP after release of NO) in culture medium under physiological conditions for 3.5 hours. Oocytes were then subjected to assays for ZPDT, spindle/chromosome morphology, CG status and OMD (23). In experiment set 3, oocytes and cumulus cells from each of the three groups were subjected to fluorescence immunocytochemistry for nitrotyrosine antibody to detect protein nitration (37).

### Superovulation and oocyte retrieval

Six to ten-week-old B6D2F1 mice obtained from Jackson Laboratories (Bar Harbor, ME) were acclimatized to the 14 h light-10 h dark cycle. The animals were housed under standard conditions until ready for experiments performed at ages 8-14 (YB), 48-52 (RB) or 80-84 weeks (OA). In all groups, animals were superovulated with 7.5 IU each of pregnant mare’s serum gonadotropin (PMSG) and human chorionic gonadotropin (hCG) respectively, 50 hours apart. In the YB and RB groups, oocytes were retrieved from oviductal ampullae at 13.5 or 17 h after hCG to obtain postovulatory young or relatively old oocytes, whereas in the OA group, oocytes were retrieved only at 13.5 h post-hCG, as the yield of already low numbers of oocytes in this age-group of animals was noted to be extremely poor. Cumuli retrieved from the oviductal ampullae were treated with 0.1% hyaluronidase (w/v) in M2 medium (Sigma) for 2-3 minutes at 37°C. Oocytes were subsequently denuded to remove all cumulus-corona cells with a narrow bore pulled glass Pasteur pipette, thoroughly rinsed in M2 medium, inspected to rule out abnormal morphology and were kept ready in M16 medium (Sigma) pre-equilibrated with 5% CO_2_ in air at 37°C in a common pool before randomly assigning oocytes into test and control groups according to the experiment sets. In experiment set 3, cumulus cells denuded from the oocytes from YB and RB retrieved at 13.5 and 17 hours after hCG were studied for NT.

### Experiment set 1

Zona pellucida dissolution time (ZPDT), was assessed for oocytes from the YB (8-14 weeks) and RB (48-52 weeks) groups retrieved at 13.5 and 17 h post-hCG and in OA, due to limited number of oocytes, at 13.5 h post-hCG group (n=156), followed by fixation in 4% paraformaldehyde (PFA), and fluorescence immunocytochemistry with α-tubulin and DAPI for spindle and chromatin morphology and fluorescent staining of cortical granules (CG) with lens culinaris agglutinin for status of cortical granules (24-26). Briefly, oocytes were subjected to acidified Tyrode’s solution to remove ZP and were fixed in 4% paraformaldehyde PFA at 37°C for 45 minutes, permeabilized using PBS Triton X-100 with 0.3% BSA for 45 minutes and blocked using 3% blocking solution overnight at 4°C and subsequently stained with primary anti α-tubulin antibody and an FITC conjugated secondary antibody as well as rhodamine conjugated pisum sativum agglutinin (PSA), and loaded in chambers containing Vectashield containing DAPI (Vector Laboratories, Eugene, OR) (24). Other oocytes were subjected to determination of ooplasmic microtubule dynamics prior to fixation and fluorescent staining (OMD, n=145) by treating with Taxol (5µM, 10 minutes, in M16 medium at 37°C with 5% CO2 in air), to selectively enhance ooplasmic microtubules in postmature, but not young, oocytes (34). Specimens were examined under confocal microscope by an independent examiner blinded to subgroups.

### Experiment set 2

Sibling M2 stage oocytes retrieved from RB at 13.5 h post-hCG were exposed to SNAP (100μM, @0.23 μM/min NO, **Table 1**) or penicillamine (M-16 medium, 37°C, 5% CO_2_, 3.5 h, **Table 1**) and were processed to examine the aging phenomena as above (ZPDT, spindle morphology, CG status and OMD (23).

**Table 1:** Oocyte groups are presented in the table indicating the hCG trigger to retrieval interval as well as subgroups used in the study design.

Oocyte Groups	hCG to Retrieval Interval	No. of Oocytes	No. of oocytes exposed to Taxol	No. of oocytes not exposed to Taxol
**YB**	13.5 h	62	30	32
**YB**	17 h	68	32	36
**RB**	13.5 h	65	32	33
**RB**	17 h	71	36	35
**OA**	13.5 h	35	15	20
**RB + SNAP**	13.5 h	30	15	15
**RB Controls**	13.5 h	30	16	14

### Experiment set 3

Oocytes and cumulus cells retrieved from the YB and RB groups were fixed after cumulus cell denudation and ZP dissolution as mentioned above, and subsequently underwent fluorescence immunocytochemistry using a rhodamine conjugated primary antibody against nitrotyrosine (NT, Cayman Chemicals, Ann Arbor, MI). Briefly, oocytes and cumulus cells were processed as follows. Oocytes were rinsed and permeabilized with phosphate buffer saline containing 0.1% triton X-100 (PBS-TX) for 1 hour at 37°C and subjected to blocking solution containing 3% BSA overnight (4°C). The oocytes were then treated with primary anti-nitrotyrosine antibody (mouse polyclonal, 1:200, Cayman Chemicals, Ann Arbor, MI) for 1 hour, rinsed with PBS-TX, treated with rhodamine conjugated secondary antibody (rabbit, anti-mouse, 1:300, Molecular probes, Eugene, OR) and mounted in glass chambers (Sigma-Aldrich). The cumulus cells were processed in a similar fashion and mounted in glass chambers. Appropriate negative controls were used (37). The processed oocytes and cumulus cells were examined under confocal microscopy by an independent examiner blinded to the subgroups. Each oocyte was scored for the presence or absence of NT staining, whereas 100 cumulus cells in each experimental replicate were scored for presence or absence of NT.

### Statistical analysis

Statistical analysis was performed using the Prism GraphPad Software, version 6 (San Diego, CA). The frequency data for OMD, CG status and spindle normality as well as presence or absence of NT staining in the oocytes and cumulus cells were analyzed using the Chi Square and Fisher’s Exact tests, while ZPDT between the subgroups in experiment sets 1 and 2 were compared using the appropriate parametric (Student’s unpaired t test for comparing two groups and one way ANOVA for three or more groups) and non-parametric tests (Mann Whitney U test for comparing between two groups and Kruskal Wallis test for comparing three or more groups) for comparison between two groups. *Post-hoc* tests were performed where appropriate. Data are expressed as mean ± SD. Significance was defined as *P*<0.05.

## Results

### Oocyte numbers and quality among subgroups

Numbers of oocytes in the YB, RB and OA groups that were assigned to specific treatment or control subgroups are presented in **Table 1**. The mean numbers of oocytes retrieved per animal underwent a significant decrease with increasing chronological age (29.8 ± 4.1 per animal in the YB group compared to 10.2 ± 2.1 in RB and 4.0 ± 1.6 in OA; *P*<0.0001). These results indicate that a significant decrease in oocyte numbers occurs with chronological age.

### Ooplasmic microtubules among oocytes from young and old animals

**Figure 2:**
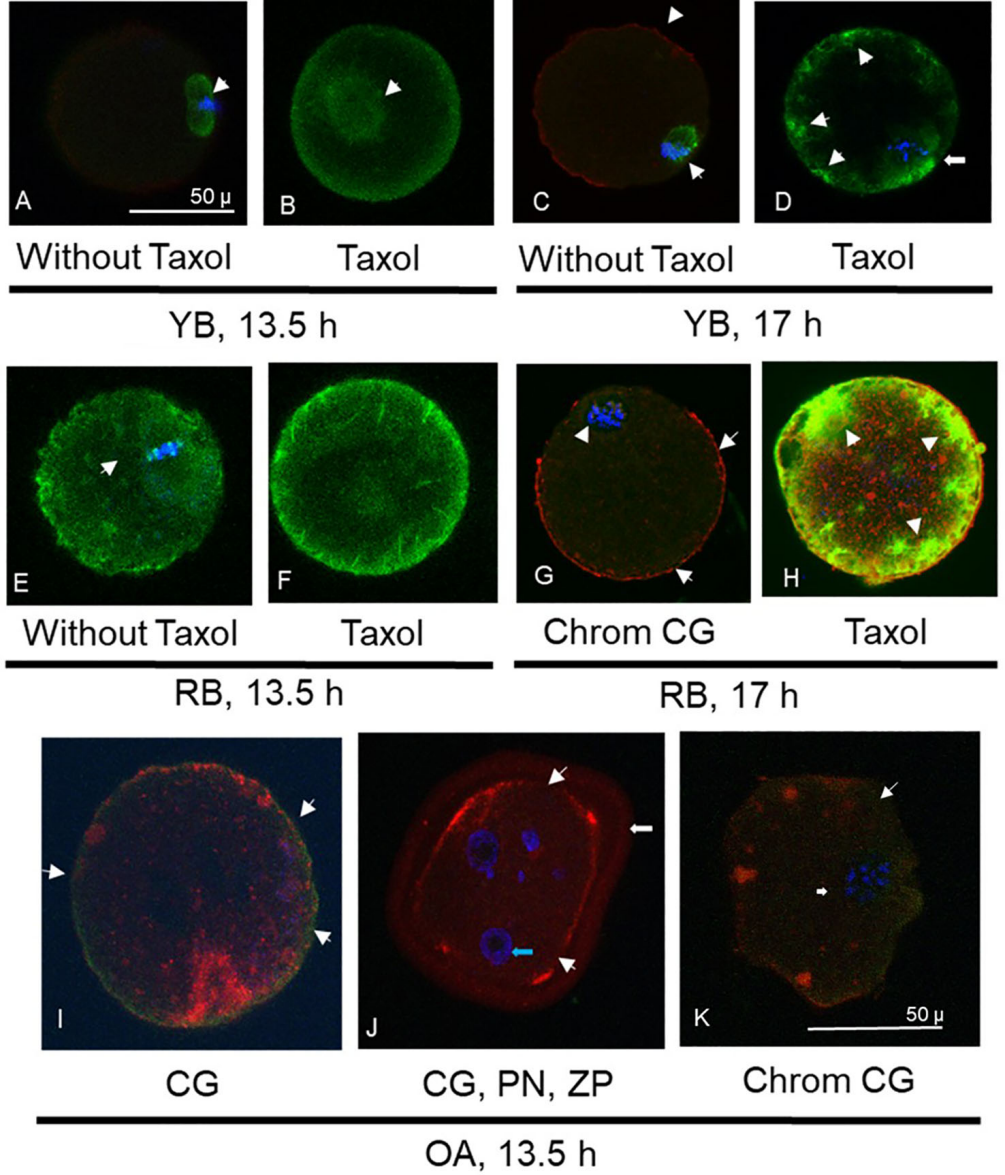
Photomicrographs are presented, depicting optical sections of oocytes labeled with fluorescent stains for α-tubulin (FITC conjugated, green, **A-H**), cortical granules (CG, rhodamine conjugated, red, **C, G-K**) and chromatin (Chrom, DAPI, blue, **A, C-E, G-K**). Photomicrographs in **(A)** and **(B)** depict spindle and ooplasmic microtubule configurations in oocytes from young breeders (YB) retrieved at 13.5 h. Arrow in **(A)** depicts normal microtubular spindle and chromosome metaphase plate. **(B)** Depicts taxol treated oocyte with enhancement of spindle microtubules (arrow) but not ooplasmic microtubules. Oocyte in **(C)** is from the YB group, retrieved at 17h post-hCG, depicting abnormal spindle morphology (arrow) and minimal cortical granule loss (arrowheads). In **(D)**, oocyte from the YB group retrieved at 17 h post-hCG has spindle irregularity as well as ooplasmic microtubule enhancement (OME), indicating effect of post-ovulatory aging. Oocytes in **(E, F)** were retrieved from RB at 13.5 h post-hCG trigger. In **(E)**, the abnormal spindle and OME are noted without taxol treatment, while in **(F)**, spindle and ooplasmic microtubule enhancement is visible (arrowheads). Oocyte in **(G)** depicts mostly intact cortical granules with minimal loss (arrows) and abnormal chromosome metaphase is seen (arrowhead). Oocyte in **(H)** was retrieved at 17 h post-hCG and shows marked enhancement of ooplasmic microtubules following taxol treatment (arrowheads). Oocytes retrieved at 13.5 h post hCG in the OA group are depicted in **(I-K)**, where major cortical granule loss is noted (arrowheads). Oocyte in **(J)** depicts pronuclear formation (PN, blue arrow) and undissolved zona pellucida (ZP, white arrow), indicating spontaneous oocyte activation and zona pellucida hardening. In **(K)**, apart from major moderate cortical granule loss (arrowheads), an abnormal chromosome metaphase plate is noted (arrow). Original magnification is 600×, bar in **(A)** and represents 50 µm, applicable to **(A-H)**, bar in **(K)** and represents 50 µm, applicable to **(I-K)**.

**Figure 3:**
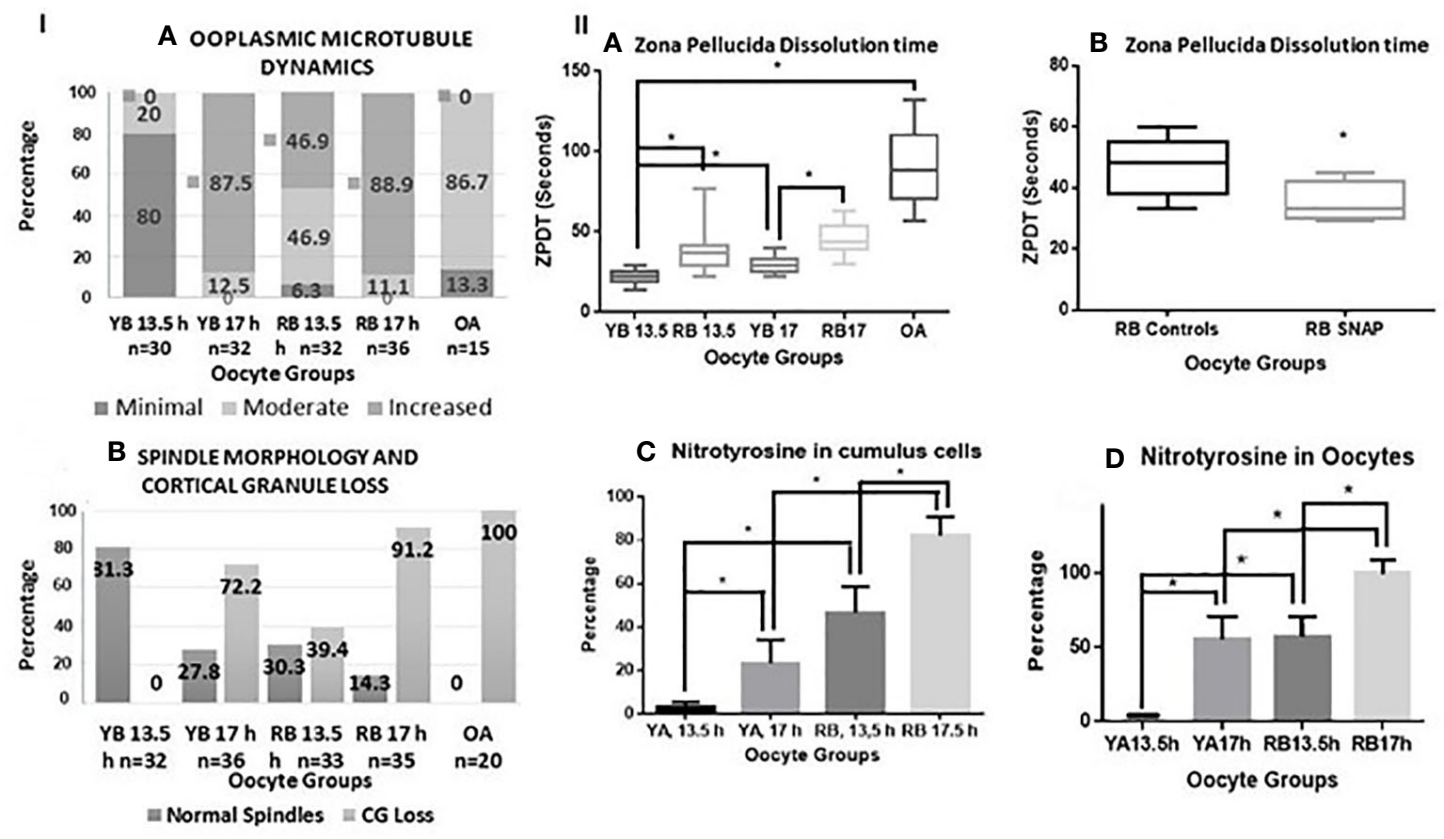
***Panel I* (A, B)** are bar charts representing categorical data presented in form of percentages. In **(A)**, percentages of oocytes with minimal, moderate, and increased ooplasmic microtubules are presented within the oocytes in the YB and RB groups, retrieved at 13.5 and 17 h post-hCG. Significant differences in mild, moderate and increased ooplasmic microtubules were noted within the YB and RB groups with respect to post-ovulatory age (minimal and increased, YB: 13.5 vs 17 h, *P*<0.0001 for both respectively; increased, RB: 13.5 h vs 17 h, *P*=0.0002; minimal and increased, YB vs RB, 13.5 h, *P*<0.0001; YB 13.5 h, RB 13.5 h and RB 17 h vs OA, *P*<0.0001 for each respectively; Moderate: YB 13.5 h vs RB 13.5 h, *P*=0.0333; RB 13.5 vs 17 h, *P*=0.013; YB 13.5 h and 17 h vs OA, *P*<0.0001 for both respectively; RB 13.5 vs OA, *P*=0.0119). In **(B)**, Percentages of oocytes with intact spindles and intact cortical granules decreased with post-ovulatory age in both YB and RB groups (P<0.0001 for both), and among oocytes retrieved at 13.5 h from YB, RB and OA groups respectively (P<0.0001). ***Panel II* (A, B)** are box-whiskers plots depicting zona pellucida dissolution time (ZPDT) in seconds on the Y axis, On the X axis in **(A)**, oocyte groups are included. YB: young breeders, RB: retired breeders, OA: Old animals. A significant increase is noted in ZPDT among oocytes retrieved at 17 h compared to 13.5 h within the YB and RB (*P*<0.0001). Similarly, a significantly higher ZPDT is noted among RB oocytes retrieved at 13.5 h and 17 h, when compared with their corresponding subgroups (13.5 and 17 h respectively) in the YB group (*P*<0.0001). **(C, D)** depict nitrotyrosine staining in cumulus cells and oocytes from YB and RB. A significant increase in NT staining is noted among oocytes retrieved at 17 h compared to 13.5 h post hCG trigger in both YB and RB groups among oocytes from RB compared to YB oocytes at 13.5 and 17 h post hCG trigger (*P*<0.0001 in all).

In YB, oocytes retrieved at 13.5 h (**Figures 2A, B**) displayed normal microtubule configurations whereas at 17 h displayed an increase in ooplasmic microtubules with visibility enhanced in the presence of Taxol (**Figures 2C, D**). Oocytes from RB showed abnormal OME with variable cortical granules (**Figures 2G, H**). It is important to note that despite the absence of Taxol (**Figure 2E**), a significant OME and an abnormal spindle are observed (**Figures 2F, G**); whereas visibility is further enhanced with Taxol exposure, indicating increased ooplasmic microtubule turnover secondary to aging events in the cytoplasm (**Figure 2H**). Similar enhancement was noted in RB oocytes at 17 h (**Figure 3** IA). For OA, oocytes were only retrieved at 13.5 h as quality measures were already reflective of post-ovulatory aging phenomena including cortical granule loss, abnormal formation of pronuclei or micronuclei, zona pellucida hardening, and abnormal spindle and microtubule status (**Figures 2I–K**).

A significant increase in ooplasmic microtubules was noted among oocytes retrieved from oocytes from YB and RB retrieved at 17 h compared to those retrieved at 13.5 h (YB: *P*<0.0001, RB: *P=*0.0002). Moreover, the number of oocytes displaying minimal, moderate and increased ooplasmic microtubules were significantly different for OA, RB and YB, overall indicating a significantly increased ooplasmic microtubules among oocytes from OA, RB and YB from highest to lowest (*P*<0.0001, **Figure 3** panel IA).

In summary, the OME increased as a function of post-ovulatory age in the YB group, whereas in the RB and OA groups, it also additionally increased as a function of chronological age.

### Spindle morphology and cortical granule status

Oocytes retrieved at 13.5 compared to 17 h had significantly higher incidence of intact microtubular spindles and CG in both YB and RB groups (*P*<0.0001). Moreover, oocytes from the YB group also had a significantly higher occurrence of intact spindles and CG compared to oocytes from the RB group at 13.5 and 17 h post-hCG and oocytes from OA group respectively (P<0.0001). A significantly higher number of oocytes from the YB and RB groups retrieved at 13.5 h displayed intact CG and normal spindles, compared to those retrieved at 17 h post-hCG (**Figure 3**, panel I B). Thus, oocytes from RB were noted to have both, pre-ovulatory as well as accelerated post-ovulatory aging; whereas the oocytes from OA displayed accelerated pre-ovulatory aging. Thus, the occurrence of normal spindle morphology and intact CG is noted to decrease with both, post-ovulatory age of the oocyte and chronological age of the animal.

### Zona pellucida hardening and spindle morphology among oocytes from young and old animals

A significant increase in ZPDT was noted with increasing post-ovulatory age in oocytes from the YB as well as the RB groups. Thus, oocytes retrieved at 17 h had significantly higher ZPDT compared to 13.5 h within the YB as well as the RB groups (*P*<0.0001). Moreover, ZPDT in oocytes retrieved at 13.5 h and 17 h post-hCG were significantly higher in the RB group compared to the oocytes retrieved at 13.5 and 17 h in the YB group (**Figure 3**, panel IIA).

All oocytes from OA displayed abnormal characteristics with respect to increased ZPDT, near total CG loss and disrupted or abnormal morphology of microtubular spindles compared to YB (**Figures 2I–L**, **3**-panel IB). Oocytes from the OA group also demonstrated spontaneous oocyte parthenogenesis, as evidenced by the incidence of either abnormal formation of pronuclei or micronuclei. This is likely a result of an unstable metaphase II spindle with scattered metaphase chromosomes, accompanied with spontaneous oocyte activation. Oocytes from OA also displayed a marked increase in cytoplasmic microtubules without taxol treatment (**Figures 2J, K**). Nonetheless, with treatment with taxol, oocytes from the OA group did not show further increase in already increased ooplasmic microtubules.

Zona pellucida hardening is noted to increase with both, postovulatory age of the oocyte and progressively with chronological age of the animal.

### Exposure to nitric oxide donor

**Figure 4:**
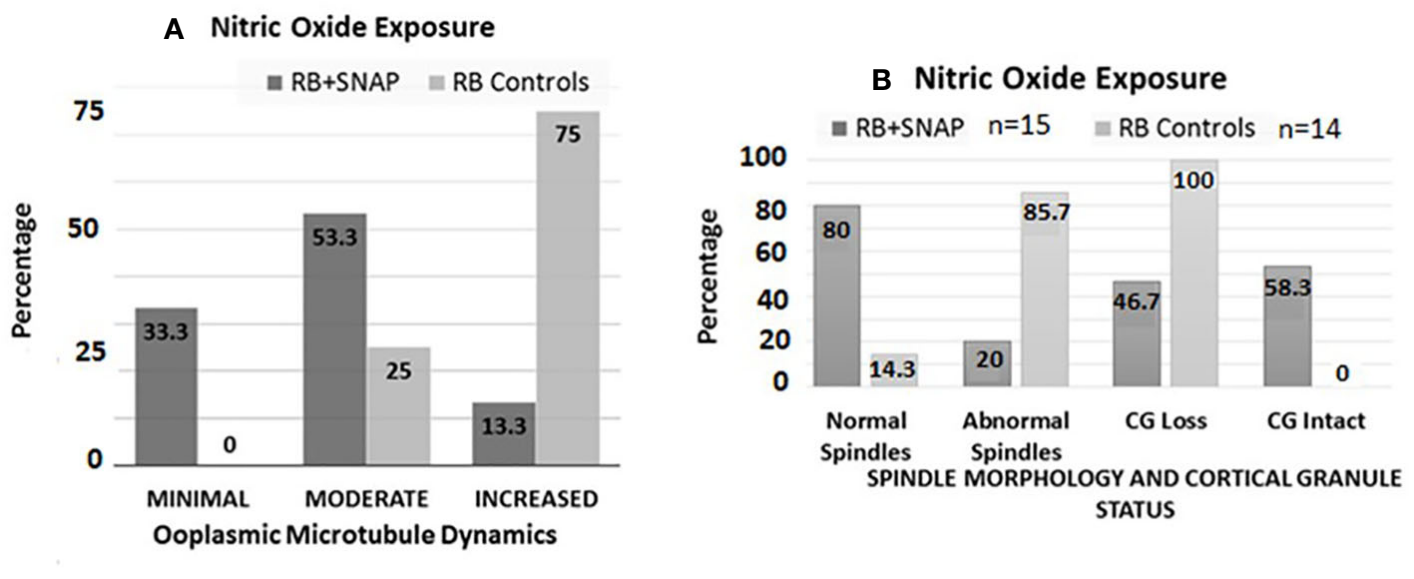
Bar chart **(A)** depicts percentages of oocytes with minimal, moderate and increased microtubule dynamics after exposure to the NO donor *S-Nitroso-N-acetyl-DL-penicillamine* (SNAP, test groups) versus penicillamine (control group). Significantly higher numbers of oocytes with minimal (*P*=0.0177), and significantly decreased numbers of oocytes with increased OMD were noted in the RB group oocytes exposed to SNAP compared to controls (*P*=0.001). Bar chart **(B)** depicts the status of spindle morphology and cortical granules in RB oocytes exposed to SNAP versus controls, similar to **(A)**. Significantly higher number of RB oocytes exposed to SNAP had normal spindle morphology compared to the control oocytes ((*P*=0.0007). RB oocytes exposed to SNAP also had significantly lower number of oocytes with cortical granule loss compared to the control oocytes (*P*=0.022).

Oocytes retrieved at 13.5 h post-hCG from the RB group exposed to SNAP had significantly lower ZPDT compared to controls exposed to penicillamine (**Figure 3**, panel II B). Furthermore, exposure to SNAP resulted in significantly higher number of oocytes with minimal (P=0.0177) and significantly decreased oocytes with increased ooplasmic microtubules despite exposure to Taxol (*P*=0.001, **Figure 4A**). Similarly, increased number of oocytes with normal spindle morphology (*P*=0.0007) and intact CG (P=0.022) were noted following exposure to SNAP versus controls (**Figure 4B**). Thus, exposure to an NO donor decreased OME, CG loss and improved spindle morphology among oocytes from RB.

### Nitrotyrosine staining

Among the YB group oocytes, nitrotyrosine (NT) staining was observed in very scant (1%) cumulus cells and in none of the oocytes retrieved at 13.5 h, but occurred in 55.5% of the oocytes retrieved at 17 h post-hCG (**Figure 3** panel II C, D, *P*<0.0001 for both). Among oocytes from the RB group, NT staining was observed in oocytes and cumulus cells retrieved at 13.5 and 17 h. However, proportion of NT-positive oocytes and cumulus cells was significantly higher in the 17 h subgroup compared to 13.5 h, and significantly higher than 17 h subgroup from the YB group. Among the oocytes from the OA group, all oocytes stained positive for NT (5 out of 5 tested, *P*<0.0001 for all). Very scant to no cumulus cells were available in the OA group and NT staining quantification or analysis was not performed for the cumulus cells in the OA group. Thus, NT positivity increased in oocytes as well as cumulus cells with advancing chronological age as well as post-ovulatory age with the highest number of NT positive oocytes and cumulus cells noted in the RB group (**Figures 3**, panel II C, D, **5**). Incidence of NT positivity in oocytes and cumulus cells thus increased progressively with both postovulatory and chronological aging.

**Figure 5:**
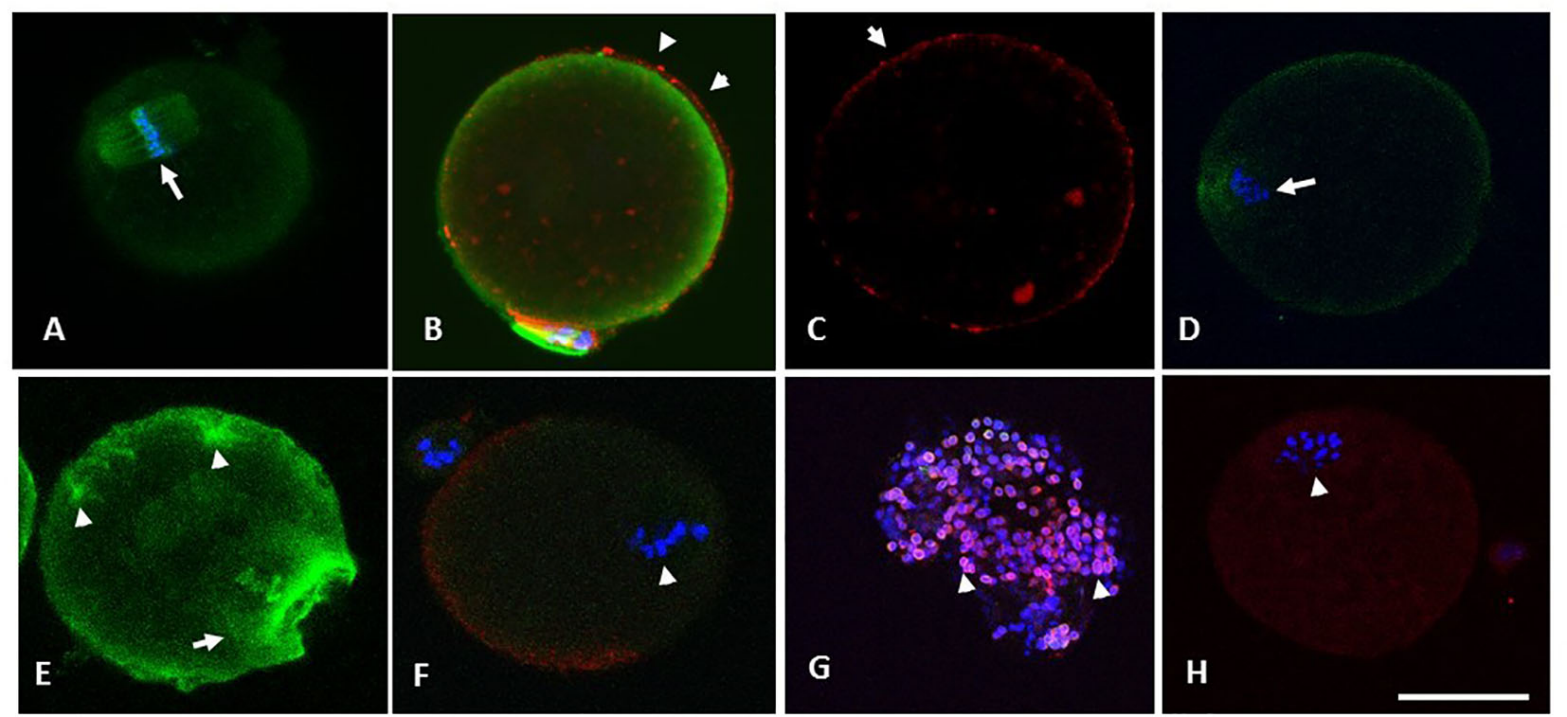
**(A)** through **(F)** are photomicrographs showing optical sections of oocytes depicting microtubules (green), chromosomes (blue) and CG (red). Oocytes in **(A)** through **(C)** were retrieved from the RB group at 13.5 h and were treated with SNAP (100 µM, 3 hours, 37°C, 5% CO_2_), whereas their sibling control oocytes were exposed to penicillamine under similar conditions **(D-F)**. Optical section **(A)** passes through the cortical cytoplasm depicting the spindle apparatus with normal morphology (arrowhead in A). Oocyte in **(B)** has minimal OMD despite treatment with Taxol. Intact CG are noted along and immediately beneath the oolemma (arrowheads in B and C). Oocyte in **(D)** shows a control oocyte untreated with NO-donor, with disrupted spindle and the metaphase plate. Similar control oocyte treated with taxol is depicted in **(E)**, with significant enhancement in ooplasmic microtubules (arrowheads in E). Photomicrograph **(F)** depicts abnormal morphology of spindle apparatus with disruption of the chromosome metaphase plates (arrow). A moderate loss of CG was noted in the same oocyte on 3-D image reconstruction, while a CG free domain is depicted. Photomicrograph **(G)** is obtained from an optical section on a specimen of cumulus cells separated from a cumulus cell-oocyte complex retrieved from the RB group at 13.5 h. The cumulus cells depict the presence of nitrotyrosine (NT, red), while the nuclei are stained with DAPI (blue). Oocyte in **(H)** is from RB, showing the presence of positive staining for NT (arrowhead depicts abnormal metaphase). Original magnification 600×, bar in H represents 50 µm.

## Discussion

Impairment in follicular dynamics with advancing age is known among mice as well as several other species, including human, in the form of lower number of recruited oocytes after superovulation (30, 38). Similarly, increased oocyte spindle abnormalities and ZP hardening events are consistent with previous observations, for example, among oocytes from cycling women with advancing age (35, 36, 39). Our study showed a serial and significant decrease in the number of oocytes with advancing age from YB to RB and OA. Moreover, an increased incidence of spindle abnormalities, CG loss and ZP hardening indicated worsening oocyte quality as a function of chronological age.

Freshly ovulated oocytes from the RB group had a significant increase in ZP hardening, premature CG loss, and increased OMD compared to YB. This was noted even at 13.5 h after ovulatory trigger, whereas under physiological conditions, these changes are typically induced only after several hours post-ovulation, e.g., at >17 h post ovulatory trigger (**Figure 3**). These events also mark the end of the physiological temporal window for optimal fertilization, as indicated by their association with fertilization and cleavage abnormalities, fragmentation as well as apoptosis (34, 35, 37). On the other hand, the oocytes retrieved from old animals not only showed signs of CG loss and ZP hardening, but also showed signs of *in-vivo* spontaneous activation, with pronuclear formation. Both phenomena noted in RB and OA are likely a continuum of the same process, well known to be related to alteration of cell cycle factors involved in meiosis exit (40). Hence, these oocytes have likely undergone alterations indicative of abolition or narrowing of the temporal window for optimal fertilization (35).

Increase in OMD in oocytes from older mice indicates a failure of sustained meiotic arrest in the M II stage. Oocytes undergo a unique meiotic cell cycle, which is arrested in the dictyate stage of prophase for an indefinite time period that can last even up to 5 decades, making them vulnerable to meiotic chromosome segregation errors during M I phase, which largely contribute to aneuploidies in the oocyte and embryo (41, 42). However, abnormality of the M II spindle and subsequent occurrence of fertilization abnormalities as well as mitotic errors in the embryo are the additional phenomena reported in older individuals (42). Stability of the M II spindle apparatus, a consequence of the stability of meiotic cell cycle factors, is critical for development of chromosomally normal embryos. Instability of the M II arrest results in a spontaneous partial meiotic exit of the oocytes due to changes in cell cycle factors. Such oocytes undergo asynchronous cytoplasmic and/or nuclear progression that is incompatible with normal development (43).

Accelerated premature meiosis transition occurring within pre-ovulatory oocytes from meiotic stage to an interphase like stage may be due to altered follicular dynamics or increased ROS, or both [ (44–47), inducing untimely meiotic exit exhibited in form of post-maturation changes. Hence, these oocytes have likely undergone alterations indicative of abolition or narrowing of the temporal window for optimal fertilization (48). At the cellular level, relatively low M phase promoting factor (MPF) and/or rapid decline in MPF activity in oocytes may cause these changes (35, 43, 48). Thus, cell cycle factor alteration is one of the key mechanisms operative in oocytes from older females contributing to poor oocyte quality noted in M II stage oocytes. It can also explain the occurrence of post-fertilization and mitotic chromosome errors, mosaicism, and developmental arrests seen for instance, in women with advanced age undergoing ART (38, 49). Furthermore, incomplete transition from meiosis to interphase may also result in ooplasmic transition to interphase while chromosomes are still in meiosis as indicated by presence of intact normal or abnormal spindle, condensed chromatin and absence of nuclear membrane, accompanied by increased microtubule array in the ooplasm (34–36). This disconnect between nuclear and cytoplasmic changes during meiotic transition can explain the occurrence of both, meiosis II and post-fertilization mitotic chromosome segregation errors, leading to embryonic aneuploidy, mosaicism, and developmental arrests seen among women with advanced age undergoing ART (38, 49). The causative mechanisms for this cell cycle advancement can be linked to failure of intracellular Ca^2+^ homeostasis and resultant elevation in cytosolic Ca^2+^, which triggers degradation of MPF (38, 48, 50, 51). Similar alterations in cell cycle factors may, therefore, explain increased incidence of spindle abnormalities among M II oocytes with advancing chronological age. This is an interesting new concept to define diminished oocyte quality related to advanced chronological age.

Our study also confirms the role of NO supplementation in slowing the post-ovulatory aging in oocytes from older mice. Furthermore, it may also be able to exert an effect on reversing pre-ovulatory aging to some degree, depending on its preexisting extent. Similar slowing of oocyte aging, with extension of the oocyte temporal window for optimal fertilization was also previously noted in our studies on NO-supplemented oocytes from younger mice as well as diabetic mice (23, 24, 37, 52, 53). Similarly, direct intracellular measurement showed significantly lower NO within oocytes from older mice (54). Therefore, NO-sufficiency is a likely mechanism operative in oocytes to both sustain their fertilization abilities and maintain quality by preventing atresia. Moreover, diminution in NO in oocytes could explain acceleration of both pre-ovulatory and post-ovulatory aging in oocytes from older mice (23, 26).

Decrease in NO levels within the oocytes and their microenvironment may occur with increasing age, due to decreased production or increased consumption of NO. Nitric oxide synthases (NOS), in the presence of molecular O_2_, tetrahydrobiopterin, (H_4_B), NADPH, flavin mononucleotide (FMN) and flavin adenine dinucleotide (FAD) convert L-arginine to citrulline, releasing NO. Insufficiency of NO may therefore result either due to deficiency of substrate or co-factors or may due to its consumption during an interaction between NO and superoxide (O_2_^•-^), resulting in the formation of highly reactive peroxynitrite (ONOO^-^), which is capable of reacting with proteins, enhancing nitration, and causing damage to cellular organelles and DNA, ultimately causing cell death (55). Furthermore, it contributes to increased protein nitration seen in the oocytes and cumulus cells from older mice in our study, in form of the nitrotyrosine footprints. Increased production of NT is therefore indicative of production of ONOO^-^ in the oocytes and their microenvironment with increasing chronological age. ONOO^-^ has been shown to augment oocyte aging (55, 56). In addition to protein nitration events, other ROS possess the capabilities to disrupt the oocyte microenvironment, without sufficient protection from the cumulus cells. ROS such as ^•^OH or HOCl have been shown to cause disturbance to the cumulus cells and directly affect the oocyte as a function of increasing concentration (25, 57, 58). Importantly, these pathological alterations can be modulated through antioxidant supplementation such as with melatonin or lycopene (59–61).

Due to their ability to enhance oocyte aging, both insufficiency of NO and increase in O_2_^•-^ are likely candidates involved in the process of accelerated oocyte meiotic transition and deterioration in oocyte quality associated with advanced chronological age. It can therefore be assumed that the role-played by ROS and NO in the oocyte and its microenvironment are the core of what drives oocyte quality deterioration. These events can be observed under circumstances of excessive ROS production including exposure to environmental toxins (62, 63), chemotherapy and drug treatments (64), gynecological diseases involving inflammation and oxidative stress (37, 65), and, as shown in this study, chronological maternal aging. As the ovarian environment is highly sensitive, systematic changes ranging from hormonal modifications to nutrition can have a direct effect on the oocyte. Moreover, the effects of chronological aging on oocytes can be accelerated due to genetic factors involved in physiological aging or epigenetic factors stemming from the environment impacting the system such as smoking, both which can disturb the oxidant-antioxidant balance resulting in over-accumulation of oxidative stress (66). Altogether, correction of the aberrant overconsumption of NO either by correcting its cause or by augmenting NO in oocytes and their microenvironment could be a potential mechanism to rectify decline in fertility and fecundability, that is invariably associated with advancing female age.

## Data availability statement

The raw data supporting the conclusions of this article will be made available by the authors, without undue reservation.

## Ethics statement

The animal study was approved by Wayne State University Institutional Animal Care and Use Committee. The study was conducted in accordance with the local legislation and institutional requirements.

## Author contributions

PG, AG, and HA-S contributed to conception and design of the study. DB and OC organized the database. DB, OC, and PG performed the statistical analysis. PG, AG, and HA-S wrote the first draft of the manuscript. OC, DB, BG, and MD wrote sections of the manuscript. All authors contributed to the article and approved the submitted version.

## References

[1] Cox CM Thoma ME Tchangalova N Mburu G Bornstein MJ Johnson CL. Infertility prevalence and the methods of estimation from 1990 to 2021: a systematic review and meta-analysis. Hum Reprod Open (2022) 2022(4):hoac051. doi: 10.1093/hropen/hoac051 36483694 PMC9725182

[2] Why American Women Everywhere Are Delaying Motherhood (2021). Available at: https://www.nytimes.com/2021/06/16/us/declining-birthrate-motherhood.html?smid=url-share.

[3] Liu K Case A. Advanced reproductive age and fertility. J Obstet Gynaecol Can (2011) 33(11):1165–75. doi: 10.1016/S1701-2163(16)35087-3 22082792

[4] Eichenlaub-Ritter U. Genetics of oocyte ageing. Maturitas (1998) 30(2):143–69. doi: 10.1016/S0378-5122(98)00070-X 9871909

[5] Faddy MJ. Follicle dynamics during ovarian ageing. Mol Cell Endocrinol (2000) 163(1-2):43–8. doi: 10.1016/S0303-7207(99)00238-5 10963872

[6] Gougeon A Ecochard R Thalabard JC. Age-related changes of the population of human ovarian follicles: increase in the disappearance rate of non-growing and early-growing follicles in aging women. Biol Reprod (1994) 50(3):653–63. doi: 10.1095/biolreprod50.3.653 8167237

[7] Kuliev A Zlatopolsky Z Kirillova I Spivakova J Cieslak Janzen J. Meiosis errors in over 20,000 oocytes studied in the practice of preimplantation aneuploidy testing. Reprod BioMed Online (2011) 22(1):2–8. doi: 10.1016/j.rbmo.2010.08.014 21115270

[8] Jones KT. Meiosis in oocytes: predisposition to aneuploidy and its increased incidence with age. Hum Reprod Update (2008) 14(2):143–58. doi: 10.1093/humupd/dmm043 18084010

[9] Treloar AE Boynton RE Behn BG Brown BW. Variation of the human menstrual cycle through reproductive life. Int J Fertil (1967) 12(1 Pt 2):77–126. 5419031

[10] Owen A Sparzak PB. Age-related fertility decline. In: StatPearls. Treasure Island (FL): StatPearls Publishing LLC (2023).

[11] Sasaki H Hamatani T Kamijo S Iwai M Kobanawa M Ogawa S. Impact of oxidative stress on age-associated decline in oocyte developmental competence. Front Endocrinol (Lausanne) (2019) 10:811. doi: 10.3389/fendo.2019.00811 31824426 PMC6882737

[12] Wang S Zheng Y Li J Yu Y Zhang W Song M. Single-cell transcriptomic atlas of primate ovarian aging. Cell (2020) 180(3):585–600.e19. doi: 10.1016/j.cell.2020.01.009 32004457

[13] Broekmans FJ Soules MR Fauser BC. Ovarian aging: mechanisms and clinical consequences. Endocr Rev (2009) 30(5):465–93. doi: 10.1210/er.2009-0006 19589949

[14] Lenton EA Landgren BM Sexton L Harper R. Normal variation in the length of the follicular phase of the menstrual cycle: effect of chronological age. Br J Obstet Gynaecol (1984) 91(7):681–4. doi: 10.1111/j.1471-0528.1984.tb04830.x 6743609

[15] te Velde ER Pearson PL. The variability of female reproductive ageing. Hum Reprod Update (2002) 8(2):141–54. doi: 10.1093/humupd/8.2.141 12099629

[16] van Zonneveld P Scheffer GJ Broekmans FJ Blankenstein MA de Jong FH Looman CW. Do cycle disturbances explain the age-related decline of female fertility? Cycle characteristics of women aged over 40 years compared with a reference population of young women. Hum Reprod (2003) 18(3):495–501. doi: 10.1093/humrep/deg138 12615813

[17] Santoro N Isaac B Neal-Perry G Adel T Weingart L Nussbaum A. Impaired folliculogenesis and ovulation in older reproductive aged women. J Clin Endocrinol Metab (2003) 88(11):5502–9. doi: 10.1210/jc.2002-021839 14602797

[18] Burger HG Dudley E Mamers P Groome N Robertson DM. Early follicular phase serum FSH as a function of age: the roles of inhibin B, inhibin A and estradiol. Climacteric (2000) 3(1):17–24. doi: 10.3109/13697130009167595 11910605

[19] Soules MR Sherman S Parrott E Rebar R Santoro N Utian W. Executive summary: stages of reproductive aging workshop (STRAW). Fertil Steril (2001) 76(5):874–8. doi: 10.1016/S0015-0282(01)02909-0 11704104

[20] Pacella-Ince L Zander-Fox DL Lan M. Mitochondrial SIRT3 and its target glutamate dehydrogenase are altered in follicular cells of women with reduced ovarian reserve or advanced maternal age. Hum Reprod (2014) 29(7):1490–9. doi: 10.1093/humrep/deu071 24771001

[21] Franasiak JM Forman EJ Hong KH Werner MD Upham KM Treff NR. The nature of aneuploidy with increasing age of the female partner: a review of 15,169 consecutive trophectoderm biopsies evaluated with comprehensive chromosomal screening. Fertil Steril (2014) 101(3):656–663.e1. doi: 10.1016/j.fertnstert.2013.11.004 24355045

[22] Hekimi S Lapointe J Wen Y. Taking a “good” look at free radicals in the aging process. Trends Cell Biol (2011) 21(10):569–76. doi: 10.1016/j.tcb.2011.06.008 PMC4074523 21824781

[23] Goud AP Goud PT Diamond MP Abu-Soud HM. Nitric oxide delays oocyte aging. Biochemistry (2005) 44(34):11361–8. doi: 10.1021/bi050711f 16114873

[24] Goud AP Goud PT Diamond MP Gonik B Abu-Soud HM. Activation of the cGMP signaling pathway is essential in delaying oocyte aging in diabetes mellitus. Biochemistry (2006) 45(38):11366–78. doi: 10.1021/bi060910e 16981697

[25] Goud AP Goud PT Diamond MP Gonik B Abu-Soud HM. Reactive oxygen species and oocyte aging: role of superoxide, hydrogen peroxide, and hypochlorous acid. Free Radic Biol Med (2008) 44(7):1295–304. doi: 10.1016/j.freeradbiomed.2007.11.014 PMC3416041 18177745

[26] Camp OG Goud AP Goud PT Bai D Awonuga A Abu-Soud HM. Diminishing oocyte quality with advancing age is associated with deficiency of nitric oxide synthase cofactors, tetrahydrobiopterin, and zinc, in mouse oocytes. F S Sci (2023) 4(2):114–20. doi: 10.1016/j.xfss.2023.02.002 36787827

[27] Abu-Soud HM Hazen SL. Nitric oxide is a physiological substrate for mammalian peroxidases. J Biol Chem (2000) 275(48):37524–32. doi: 10.1074/jbc.275.48.37524 11090610

[28] Faddy MJ Gosden RG Edwards RG. Ovarian follicle dynamics in mice: a comparative study of three inbred strains and an F1 hybrid. J Endocrinol (1983) 96(1):23–33. doi: 10.1677/joe.0.0960023 6822780

[29] Tatone C Amicarelli F Carbone MC Monteleone P Caserta D Marci R. Cellular and molecular aspects of ovarian follicle ageing. Hum Reprod Update (2008) 14(2):131–42. doi: 10.1093/humupd/dmm048 18239135

[30] Coxworth JE Hawkes K. Ovarian follicle loss in humans and mice: lessons from statistical model comparison. Hum Reprod (2010) 25(7):1796–805. doi: 10.1093/humrep/deq136 20504871

[31] Yamada-Fukunaga T Yamada M Hamatani T Chikazawa N Ogawa S Akutsu H. Age-associated telomere shortening in mouse oocytes. Reprod Biol Endocrinol (2013) 11:108. doi: 10.1186/1477-7827-11-108 24261933 PMC3842639

[32] Kikuchi K Naito K Noguchi J Shimada A Kaneko H Yamashita M. Maturation/M-phase promoting factor: a regulator of aging in porcine oocytes. Biol Reprod (2000) 63(3):715–22. doi: 10.1095/biolreprod63.3.715 10952912

[33] Solon-Biet SM Walters KA Simanainen UK McMahon AC Ruohonen K Ballard JW. Macronutrient balance, reproductive function, and lifespan in aging mice. Proc Natl Acad Sci U.S.A. (2015) 112(11):3481–6. doi: 10.1073/pnas.1422041112 PMC4371964 25733862

[34] Goud AP Goud PT Van Oostveldt P Diamond MP Dhont M. Dynamic changes in microtubular cytoskeleton of human postmature oocytes revert after ooplasm transfer. Fertil Steril (2004) 81(2):323–31. doi: 10.1016/j.fertnstert.2003.06.033 14967368

[35] Goud AP Goud PT Diamond MP Van Oostveldt P Hughes MR. Microtubule turnover in ooplasm biopsy reflects ageing phenomena in the parent oocyte. Reprod BioMed Online (2005) 11(1):43–52. doi: 10.1016/S1472-6483(10)61297-7 16102286

[36] Battaglia DE Goodwin P Klein NA Soules MR. Influence of maternal age on meiotic spindle assembly in oocytes from naturally cycling women. Hum Reprod (1996) 11(10):2217–22. doi: 10.1093/oxfordjournals.humrep.a019080 8943533

[37] Goud PT Goud AP Joshi N Puscheck E Diamond MP Abu-Soud HM. Dynamics of nitric oxide, altered follicular microenvironment, and oocyte quality in women with endometriosis. Fertil Steril (2014) 102(1):151–159.e5. doi: 10.1016/j.fertnstert.2014.03.053 24825428

[38] Yamamoto T Iwata H Goto H Shiratuki S Tanaka H Monji Y. Effect of maternal age on the developmental competence and progression of nuclear maturation in bovine oocytes. Mol Reprod Dev (2010) 77(7):595–604. doi: 10.1002/mrd.21188 20575084

[39] Longo FJ. Changes in the zones pellucidae and plasmalemma of aging mouse eggs. Biol Reprod (1981) 25(2):399–411. doi: 10.1095/biolreprod25.2.399 7306632

[40] Eichenlaub-Ritter U. Oocyte ageing and its cellular basis. Int J Dev Biol (2012) 56(10-12):841–52. doi: 10.1387/ijdb.120141ue 23417406

[41] Jones KT Lane SI. Molecular causes of aneuploidy in mammalian eggs. Development (2013) 140(18):3719–30. doi: 10.1242/dev.090589 23981655

[42] Vogt E Kirsch-Volders M Parry J Eichenlaub-Ritter U. Spindle formation, chromosome segregation and the spindle checkpoint in mammalian oocytes and susceptibility to meiotic error. Mutat Res (2008) 651(1-2):14–29. doi: 10.1016/j.mrgentox.2007.10.015 18096427

[43] Xu Z Abbott A Kopf GS Schultz RM Ducibella T. Spontaneous activation of ovulated mouse eggs: time-dependent effects on M-phase exit, cortical granule exocytosis, maternal messenger ribonucleic acid recruitment, and inositol 1,4,5-trisphosphate sensitivity. Biol Reprod (1997) 57(4):743–50. doi: 10.1095/biolreprod57.4.743 9314575

[44] Perkins AT Das TM Panzera LC Bickel SE. Oxidative stress in oocytes during midprophase induces premature loss of cohesion and chromosome segregation errors. Proc Natl Acad Sci U.S.A. (2016) 113(44):E6823–e6830. doi: 10.1073/pnas.1612047113 27791141 PMC5098651

[45] Jo YJ Yoon SB Park BJ Lee SI Kim KJ Kim SY. Particulate matter exposure during oocyte maturation: cell cycle arrest, ROS generation, and early apoptosis in mice. Front Cell Dev Biol (2020) 8:602097. doi: 10.3389/fcell.2020.602097 33324650 PMC7726243

[46] Perkins AT Greig MM Sontakke AA Peloquin AS McPeek MA Bickel SE. Increased levels of superoxide dismutase suppress meiotic segregation errors in aging oocytes. Chromosoma (2019) 128(3):215–22. doi: 10.1007/s00412-019-00702-y PMC6823651 31037468

[47] Morado S Cetica P Beconi M Thompson JG Dalvit G. Reactive oxygen species production and redox state in parthenogenetic and sperm-mediated bovine oocyte activation. Reproduction (2013) 145(5):471–8. doi: 10.1530/REP-13-0017 23630331

[48] Tian XC Lonergan P Jeong BS Evans AC Yang X. Association of MPF, MAPK, and nuclear progression dynamics during activation of young and aged bovine oocytes. Mol Reprod Dev (2002) 62(1):132–8. doi: 10.1002/mrd.10072 11933170

[49] Capalbo A Bono S Spizzichino L Biricik A Baldi M Colamaria S. Sequential comprehensive chromosome analysis on polar bodies, blastomeres and trophoblast: insights into female meiotic errors and chromosomal segregation in the preimplantation window of embryo development. Hum Reprod (2013) 28(2):509–18. doi: 10.1093/humrep/des394 23148203

[50] Takahashi T Igarashi H Amita M Hara S Matsuo K Kurachi H. Molecular mechanism of poor embryo development in postovulatory aged oocytes: mini review. J Obstet Gynaecol Res (2013) 39(10):1431–9. doi: 10.1111/jog.12111 23876057

[51] Tarín JJ Pérez-Albalá S Cano A. Consequences on offspring of abnormal function in ageing gametes. Hum Reprod Update (2000) 6(6):532–49. doi: 10.1093/humupd/6.6.532 11129687

[52] Goud PT Goud AP Diamond MP Gonik B Abu-Soud HM. Nitric oxide extends the oocyte temporal window for optimal fertilization. Free Radic Biol Med (2008) 45(4):453–9. doi: 10.1016/j.freeradbiomed.2008.04.035 PMC3786211 18489913

[53] Devine PJ Perreault SD Luderer U. Roles of reactive oxygen species and antioxidants in ovarian toxicity. Biol Reprod (2012) 86(2):27. 22034525 10.1095/biolreprod.111.095224 PMC3290661

[54] Goud PT Goud AP Najafi T Gonik B Diamond MP Saed GM. Direct real-time measurement of intra-oocyte nitric oxide concentration in vivo. PloS One (2014) 9(6):e98720. doi: 10.1371/journal.pone.0098720 24887331 PMC4041775

[55] Banerjee J Shaeib F Maitra D Saed GM Dai J Diamond MP. Peroxynitrite affects the cumulus cell defense of metaphase II mouse oocytes leading to disruption of the spindle structure in vitro. Fertil Steril (2013) 100(2):578–84.e1. doi: 10.1016/j.fertnstert.2013.04.030 23721714

[56] Khan SN Shaeib F Thakur M Jeelani R Awonuga AO Goud PT. Peroxynitrite deteriorates oocyte quality through disassembly of microtubule organizing centers. Free Radic Biol Med (2016) 91:275–80. doi: 10.1016/j.freeradbiomed.2015.12.033 26746586

[57] Shaeib F Khan SN Ali I Thakur M Saed MG Dai J. The defensive role of cumulus cells against reactive oxygen species insult in metaphase II mouse oocytes. Reprod Sci (2016) 23(4):498–507. doi: 10.1177/1933719115607993 26468254 PMC5933187

[58] Shaeib F Banerjee J Maitra D Diamond MP Abu-Soud HM. Impact of hydrogen peroxide-driven Fenton reaction on mouse oocyte quality. Free Radic Biol Med (2013) 58:154–9. doi: 10.1016/j.freeradbiomed.2012.12.007 PMC4482232 23261938

[59] Pennathur S Maitra D Byun J Sliskovic I Abdulhamid I Saed GM. Potent antioxidative activity of lycopene: A potential role in scavenging hypochlorous acid. Free Radic Biol Med (2010) 49(2):205–13. doi: 10.1016/j.freeradbiomed.2010.04.003 PMC3416054 20388538

[60] Khan SN Shaeib F Najafi T Kavdia M Gonik B Saed GM. Diffused intra-oocyte hydrogen peroxide activates myeloperoxidase and deteriorates oocyte quality. PloS One (2015) 10(7):e0132388. doi: 10.1371/journal.pone.0132388 26197395 PMC4511228

[61] Shaeib F Khan SN Thakur M Kohan-Ghadr HR Drewlo S Saed GM. The impact of myeloperoxidase and activated macrophages on metaphase II mouse oocyte quality. PloS One (2016) 11(3):e0151160. doi: 10.1371/journal.pone.0151160 26982351 PMC4794194

[62] Yahfoufi ZA Bai D Khan SN Chatzicharalampous C Kohan-Ghadr HR Morris RT. Glyphosate induces metaphase II oocyte deterioration and embryo damage by zinc depletion and overproduction of reactive oxygen species. Toxicology (2020) 439:152466. doi: 10.1016/j.tox.2020.152466 32315717

[63] Jeelani R Chatzicharalampous C Kohan-Ghadr HR Awonuga A Joshi N Morris RT. Acrolein, a commonly found environmental toxin, causes oocyte mitochondrial dysfunction and negatively affects embryo development. Free Radic Res (2018) 52(9):929–38. doi: 10.1080/10715762.2018.1487559 29886754

[64] Jeelani R Khan SN Shaeib F Kohan-Ghadr HR Aldhaheri SR Najafi T. Cyclophosphamide and acrolein induced oxidative stress leading to deterioration of metaphase II mouse oocyte quality. Free Radic Biol Med (2017) 110:11–8. doi: 10.1016/j.freeradbiomed.2017.05.006 PMC6854673 28499912

[65] Thakur M Shaeib F Khan SN Kohan-Ghadr HR Jeelani R Aldhaheri SR. Galactose and its metabolites deteriorate metaphase II mouse oocyte quality and subsequent embryo development by disrupting the spindle structure. Sci Rep (2017) 7(1):231. doi: 10.1038/s41598-017-00159-y 28331195 PMC5427935

[66] Brenner CA Nichols SM Jacoby ES Bavister BD. Non-human primates as a model for reproductive aging and human infertility. Gynecol Obstet Invest (2004) 57(1):21–3. 14971419

